# Left ventricular strain values using 3D speckle-tracking echocardiography in healthy adults aged 20 to 72 years

**DOI:** 10.1007/s10554-020-02100-3

**Published:** 2020-11-23

**Authors:** Ferit Onur Mutluer, Daniel J. Bowen, Roderick W. J. van Grootel, Jolien W. Roos-Hesselink, Annemien E. Van den Bosch

**Affiliations:** 1 Department of Cardiology, Erasmus University Medical Center, P.O. Box 2040, 3000 CA Rotterdam, The Netherlands; 2 Department of Cardiology, Erasmus University Medical Center, Room Rg-433,‘s-Gravendijkwal 230, 3015 CE Rotterdam, The Netherlands

**Keywords:** Speckle-tracking, Strain, Echocardiography, Three-dimensional, Left ventricle, Healthy subjects

## Abstract

Three dimensional speckle tracking echocardiography (3D-STE) is a novel modality for the assessment of left ventricular strain (LVS). The aim of our study is to provide single vendor normative strain values measured with 3D-STE in healthy adult caucasians. One hundred fifty-five healthy subjects aged 20 to 72 years (≥ 28 subjects per decile) were prospectively included and examined with 2D and 3D transthoracic echocardiography. In 105 both 3D and 2D-STE were feasible (71%, mean age 44 ± 14 years, 51% female). Mean 3D tangential strain (3D-TS) was − 32 ± 2.9%. 3D global longitudinal strain (3D-GLS) demonstrated a significant but not very strong correlation with 2D-GLS values (− 19.7 ± 1.8% vs − 20.4 ± 2.2%, r = 0.462, p < 0.001). No gender difference was observed in 3D strain parameters. 3D-GLS decreased with increasing age stratum (p = 0.024). LVEF was associated with 3D-TS and 3D-GLS (r =  − 0.819, p ≤ 0.001 for 3D-TS, p =  − 0.477, r < 0.001, p = 0.001 for 3D-GLS). In this single vendor study age and gender-specific normative LV 3D-TS values were reported for healthy adult caucasians. In a significant proportion of the subjects 3D-STE was not feasible, but when feasible, 3D-STE shows excellent association with LVEF, and is therefore a promising novel modality for the assessment of the myocardial function, provided that issues of limited feasibility and temporal resolution are addressed.

## Introduction

Speckle-tracking echocardiography (STE) is a novel imaging modality that allows quantitative assessment of global and segmental left ventricular (LV) myocardial function by measuring LV strain (LVS) in a manner largely independent of angle and ventricular geometry [1]. Myocardial deformation measurements allow early diagnosis of LV dysfunction by facilitating better risk stratification, reclassification and treatment in patients with cardiovascular disease [2]. 2D-STE, which is the established echocardiographic modality for LVS analysis, has the inherent limitations of planar simplification and apical foreshortening [3].

**Fig. 1:**
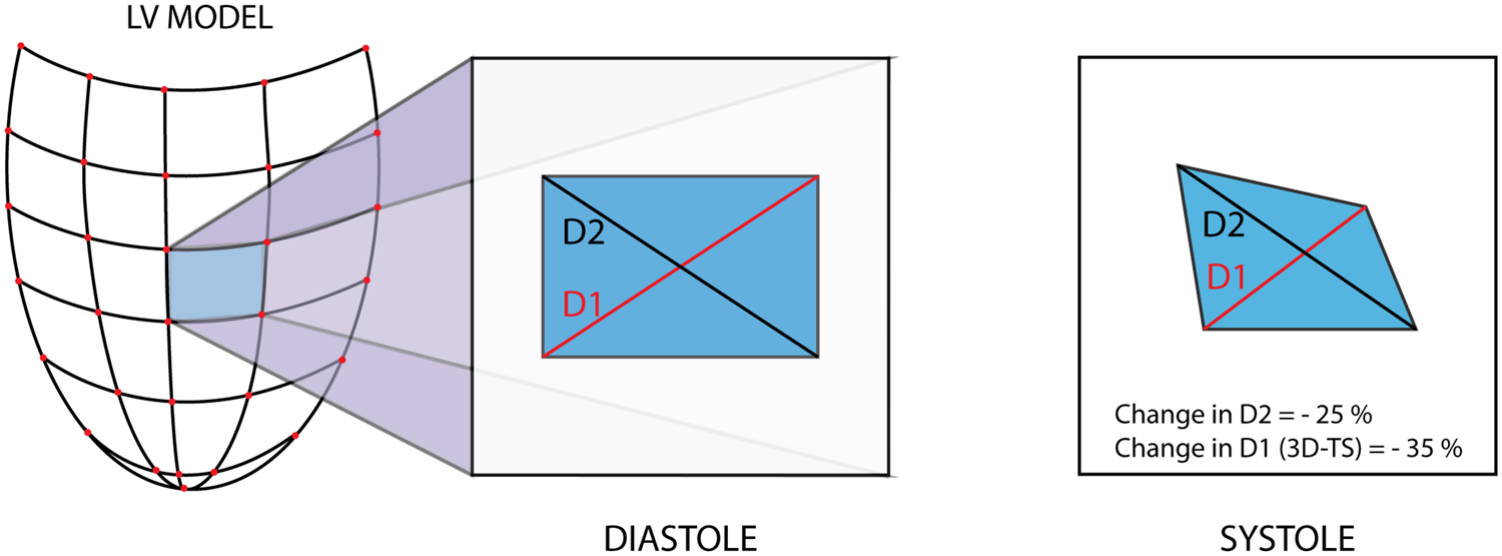
LV model: Software generated polygon mesh of the LV endocardial surface formed by connecting 16 longitudinal and 32 circumferential vertices (red dots) with edges (connecting lines). *D1 and D2* diagonals of an endocardial polygon unit, *3D-TS* tangential strain

3D-STE has been developed with the aim of overcoming the technical limitations associated with 2D-STE. This technique allows tracking of speckle patterns that move out of the imaging plane. A novel myocardial deformation parameter, 3D tangential strain (3D-TS), which is defined as the greater of the 2 deformation values along the 2 diagonals of the quadrangular endocardial units formed by the analysis software (Fig. 1),could also be calculated with this modality [4]. Several studies, the largest being the NORRE study by the European Association of Cardiovascular Imaging (EACVI), have evaluated the LVS patterns by 3D-STE in healthy adults. Reference ranges in healthy adults were proposed, and age and gender-related distribution patterns of these parameters were described [5, 6].

The previous studies demonstrated significant differences in LVS values. These differences are attributed to the ultrasound (US) system [7], STE analysis software [8], and the population studied [5]. A recent meta-analysis demonstrated that both software vendor, US system and the population studied were independently associated with the variability in the LVS values measured by 3D-STE [6]. The NORRE study, the largest cohort of 3D-STE data on healthy individuals, used the Tomtec software (Tomtec, TomTec Imaging Systems, Unterschleissheim, Germany) for STE analysis. Two different US systems, Philips (Philips Medical Systems, Best, the Netherlands) and GE (GE Vingmed Ultrasound AS, Horten, Norway), were used for image acquisition in this study. The selected image acquisition and STE analysis system varies from study to study, and makes the comparability and interpretation of the results difficult. There is a need for population specific, single US system and single analysis software data on healthy individuals.

In the current study, our aim was threefold: (1) to provide normative values for 3D-STE in a cohort of healthy adult caucasians aged 20–72 years, obtained with a single-vendor US (Philips) and STE analysis platform (Tomtec) (2) to compare the feasibility and the distribution of the 2D and 3D-STE derived LVS; (3) and to evaluate the association of 3D-STE derived LVS patterns with baseline characteristics of the study participants.

## Materials and methods

### Study population

This prospective cohort study was conducted in 2014–2015 in 155 healthy volunteers aged 20 to 72 years, who were stratified into 5 age groups: 20 to 29, 30 to 39, 40 to 49, 50 to 59, and 60 to 72 years (n ≥ 28 in each group, equal distribution in sex). The subjects were recruited via an advertisement for healthy subjects. The inclusion criteria required that subjects had normal results on physical examination and electrocardiography (ECG). Subjects were excluded when they met any of the following criteria: prior cardiovascular disease; cardiovascular risk factors consisting of hypertension, diabetes mellitus or hypercholesterolemia; systemic disease or medication known to influence cardiac function; or the finding of cardiac abnormalities during examination. Professional athletes, morbidly obese subjects (body mass index, BMI > 40 kg/m^2^), pregnant women and women with breast implants were also excluded.

### Clinical assessment

The assessment consisted of physical examination, 12-lead ECG, and echocardiography. Physical examination included height, weight, blood pressure, saturation, and results of heart, lungs and abdominal findings.

### Echocardiographic image acquisition

All echocardiographic studies were performed by 2 experienced sonographers. Two-dimensional greyscale harmonic images were obtained in the left lateral decubitus position using an iE33 or EPIQ7 ultrasound system (Philips Medical Systems, Best, the Netherlands) equipped with a transthoracic broadband X5-1 matrix transducer (composed of 3040 elements with 1–5 MHz). Standard apical 4-chamber (A4C), 2-chamber (A2C) and 3-chamber (A3C) views were obtained for STE at frame rates of ≥ 50 frames/sec. Real-time 3D-TTE was performed immediately after the 2D-TTE with the same ultrasound unit and transducer. A minimum 4-beat full volume dataset of the LV was acquired from the apical window during a single breath hold. The acquired images were transferred to the analysis software on a dedicated workstation. Conventional echocardiographic measurements including detailed left atrial mitral inflow and pulmonary vein inflow measurements were performed using the proprietary software of the echocardiography machine [9–11]. 2D-STE and 3D-STE analyses were performed using Image Arena version 4.6 (TomTec Imaging Systems, Unterschleissheim, Germany) by 2 investigators experienced in the field (FOM) and (DB). The data sets were blinded for the observers. The average peak endocardial strain values given by the software were exported to a spreadsheet program.

### Conventional echocardiographic measurements

We used the current guidelines of the American Society of Echocardiography and EACVI for chamber quantification [12]. From the A4C, pulsed-wave Doppler examination was performed to obtain peak mitral inflow velocities at early (E) and late (A) diastole and E-wave deceleration time (DT). Tissue Doppler imaging (TDI) was performed to obtain myocardial tissue velocity at the septal mitral annulus at early diastole (E’). 2D LV end-diastolic volume (LVEDV), LV end-systolic volume (LVESV) and LVEF were derived with the biplane method of disks based on one cardiac cycle.

### 2D-STE strain analysis

2D Cardiac Performance Analysis package of the Tomtec software was used for 2D-STE. The 2D-STE was defined as feasible when all the strain parameters were retrievable and the number of poorly tracking or non-tracking segments was ≤ 2. Cardiac cycles were defined by the position of the R-waves. End-diastole was tagged at the frame corresponding to peak of R wave. End-systole was tagged at the first frame at which LV has the minimal size following the reference R wave. The endocardial borders were manually traced in the A4C, A3C and A2C for the LS and in the mid-papillary level short-axis images for radial and circumferential strain. After the manual tracing, the software automatically tracked the endocardial border. Manual corrections were made when necessary. Peak systolic strain values were defined as the peak values on the curves during the ejection phase of one cardiac cycle. The 2D average global longitudinal strain, mid-papillary level average circumferential and radial strain (2D-MS-CS, 2D-MS-RS), LVEDV, LVESV and LVEF values were obtained.

### 3D-STE strain analysis

**Fig. 2:**
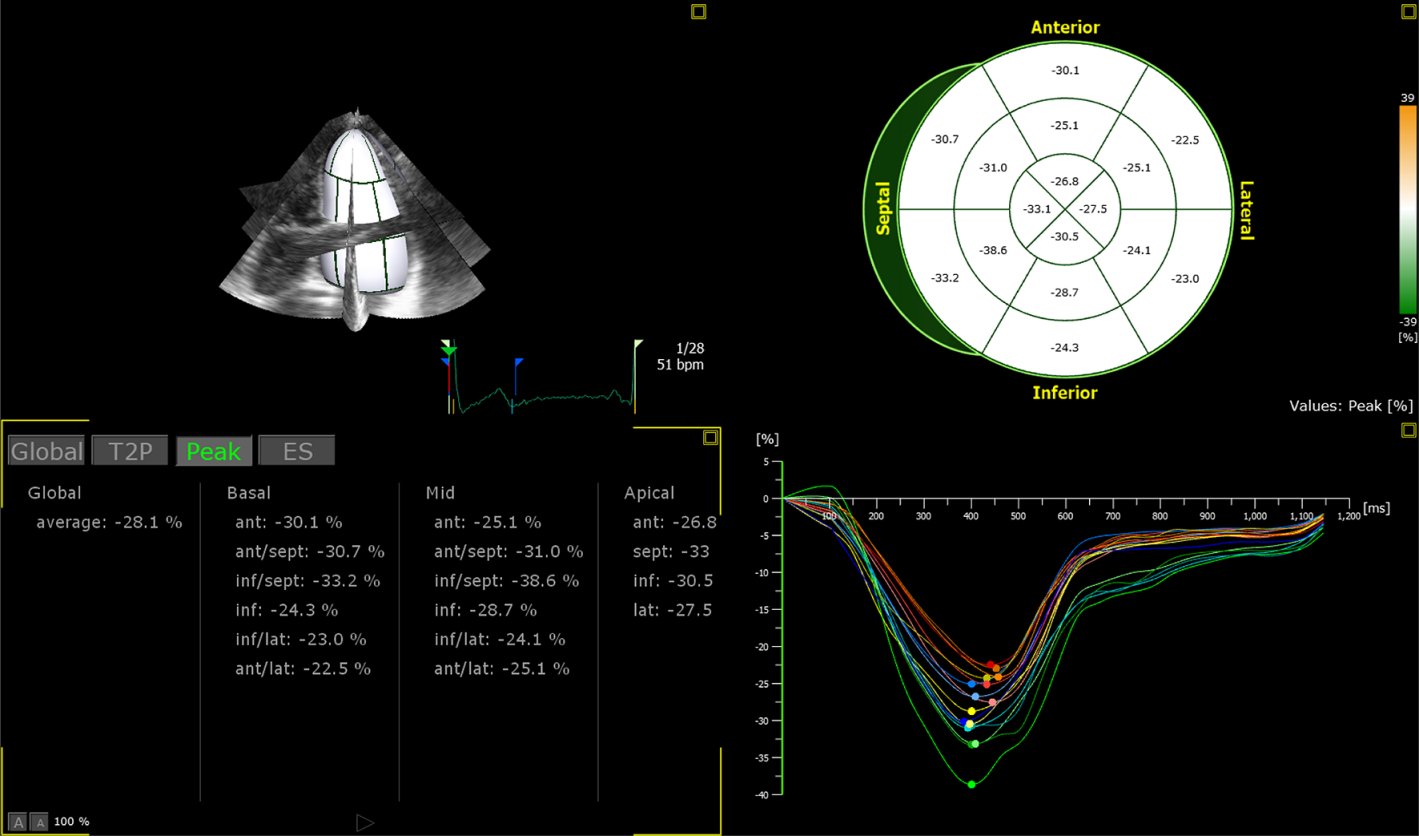
Evaluation of 3D-TS with the use of Tomtec 4D-LV Quantification package. The global average 3D-TS value in this patient was − 28.1%

4D LV-analysis package of the Tomtec software was used for 3D-STE analysis. The software processed the 3D full volume dataset and produced the 2, 3 and 4 chamber views automatically. In the respective panels displaying these views, an axial line segment was aligned by the operator manually so that it was positioned between the center point of the mitral annulus and the LV apex. The software automatically traced the LV endocardium frame-by-frame in the next step. Manual corrections were made on the automatically traced endocardial borders as needed, in the end-systolic and end-diastolic frames. Finally, the software gave the peak and end-systolic 3D-TS, 3D-GLS, 3D-GCS, 3D-GRS, twist and torsion values as well as 3D-LVEDV, 3D-LVESV and 3D-LVEF. The analysis software derives the radial strain measurements from the endocardial area, according to the principle of volume conservation. Circumferential strain is derived from the endocardial border [13]. Tracking quality was classified as excellent, good or fair, and the signal to noise ratio was classified as good or bad, subjectively by the operator. 3D-STE analysis was defined as feasible when all the strain parameters were retrievable and the number of poorly tracking or non-tracking segments was ≤ 2 (Fig. 2) [4].

### Statistical analysis

The data distribution was tested using histograms and the Shapiro–Wilk test. Continuous data were presented as mean ± standard deviation (SD). Categorical data was presented as frequencies and percentages. For comparison of normally distributed continuous between two groups with the Student’s *t*-test. In case of skewed distribution, the Mann–Whitney-U test was applied instead of t-test. For comparison of frequencies the χ^2^-test was used. For comparison of distribution of continuous variables between age strata, one-way ANOVA test was used. Analysis of correlation was performed with Pearson’s correlation coefficient. Multiple linear regression analysis was used for determining independent effect of predictors which were included in the prediction model. Two-tailed tests were used for correlation analysis and complete case analysis was the preferred method.

Intra-observer agreement was assessed by repeated analysis in a sample of 20 subjects (n = 4 per decile, randomly selected) > 1 month after the initial analysis, in randomly mixed order, and blinded to the initial results. Assessment of inter-observer agreement was performed by the second investigator (DB) in the same sample. The agreement between 2 measurements was determined by correlation analysis with scatter plots and Bland–Altman analysis with Bland–Altman plots for determination of intra and inter-observer variabilities. All statistical analyses were performed using SPSS statistics version 25 (IBM Corp., Armonk, NY, USA). A p value of less than 0.05 was considered statistically significant.

## Results

### Study population

Of the 155 subjects who came to our medical center, 147 were included in this study (age 45 ± 14 years, 50.3% female). Eight subjects were excluded due to limited echocardiographic acoustic windows (n = 2), valve pathology (n = 2), surgically closed patent ductus arteriosus (n = 1), hypertension (n = 1), morbid obesity (n = 1), and right bundle branch block on ECG (n = 1) [9]. Table 1 shows the characteristics of the study population.

**Table 1:** Characteristics of the study population

Characteristic	All patients	Patients in whom 3D and 2D-STE are both feasible		P
	(n = 147)	Yes (n = 105)	No (n = 42)	
Sex, female	74 (50.3%)	56 (50.9%)	18 (48.6%)	0.277
Age (years)	45 ± 14	44 ± 14	47 ± 14	0.140
Current smoker	13 (8.8%)	8 (7.6%)	5 (11.9%)	0.520
Physical examination				
Body mass index (kg/m^2^)	24.4 ± 3.3	**23.9 ± 2.9**	**25.5 ± 3.9**	**0.008**
Body surface area (m^2^)	1.89 ± 0.19	1.88 ± 0.19	1.93 ± 0.20	0.104
Systolic blood pressure (mmHg)	127 ± 15	126 ± 14	129 ± 15	0.287
Diastolic blood pressure (mmHg)	80 ± 9	79 ± 9	80 ± 9	0.569
ECG				
Sinus rhythm	147 (100%)	110 (100%)	37 (100%)	–
Heart rate (bpm)	62 ± 10	61 ± 10	62 ± 9	0.741
PR interval (ms)	159 ± 22	158 ± 23	160 ± 20	0.726
QRS duration (ms)	96 ± 9	96 ± 9	96 ± 10	0.855
Echocardiography, left ventricle				
EDD (mm)	45 ± 4	45 ± 4	46 ± 4	0.354
ESD (mm)	28 ± 4	28 ± 4	28 ± 5	0.610
E wave (m/s)	0.69 ± 0.16	**0.72 ± 0.16**	**0.62 ± 0.13**	** < 0.001**
A wave (m/s)	0.49 ± 0.15	0.49 ± 0.15	0.50 ± 0.16	0.688
Deceleration time (ms)	190 ± 41	188 ± 40	195 ± 42	0.357
E/E'-ratio	8 ± 2	8 ± 2	8 ± 2	0.739
LVEDV (mL)^a^	119 ± 26	118 ± 26	120 ± 24	0.765
LVESV (mL)^a^	47 ± 13	47 ± 14	49 ± 13	0.289
LVEDVI (mL/m^2^)^a^	62 ± 10	63 ± 10	62 ± 9	0.628
LVESVI (mL/m^2^)^a^	25 ± 6	25 ± 6	25 ± 5	0.502
LVEF, biplane (%)^a^	60 ± 5	**61 ± 5**	**59 ± 4**	**0.033**

Bold correspond to the parameters with values with a significant difference between comparison groups (P < 0.05)

*LV* left ventricle, *EDD* end-diastolic diameter, *ESD* end-systolic diameter, *ESV* end-systolic volume, *EDV* end-diastolic volume, *ESVI and EDVI* values indexed to body surface area. Values are presented as n (%) or mean ± SD. *E* peak mitral inflow velocity at early diastole, *A* peak mitral inflow velocity at late diastole, E’ = early diastolic annular myocardial velocity, *LV* = left ventricle, *BSA* body surface area

^a^Volumes and ejection fraction in this table are calculated by biplane method of disks

### Feasibility, temporal resolution and image quality

3D-STE was feasible in 117 of the 147 participants (79.6%) while 2D-STE was feasible in 130 participants (88.4%) (p < 0.001). When the patients in whom 3D-STE was feasible and non-feasible were compared, 3D-STE feasible participants had a greater BMI and a lower E-wave amplitude (Table 1). In 105 participants (71.4%) both 3D and 2D-STE were feasible and were included in the further analyses. Frame rates of the echocardiographic images were lower for 3D-STE compared with 2D-STE (30 ± 10 vs 60 ± 11 Hz, p < 0.001). Majority of the participants demonstrated good tracking quality (n = 58, 55.2%) and good signal to noise ratio (n = 78, 74.3%). Fourty-nine participants (47%) had at least one poorly tracked or non-tracked segments. The segments commonly associated with tracking problems were basal antero-septum, infero-septum and infero-lateral segments (Table 2).

**Table 2:** Three-dimensional speckle tracking echocardiography image quality

	n = 105
Tracking quality	
Excellent	33 (31.4%)
Good	58 (55.2%)
Fair	14 (13.3%)
Signal to noise ratio	
Good	78 (74.3%)
Bad	27 (25.7%)
Non-tracking or poorly tracking segments	
Basal antero-septum	26 (24.8%)
Basal infero-septum	26 (24.8%)
Basal infero-lateral	6 (5.7%)
Number of non-tracking or poorly tracking segments	
0	56 (53.3%)
1	25 (23.8%)
2	24 (22.9%)

Values are presented as n (%) or mean ± SD

### LV strain

**Fig. 3:**
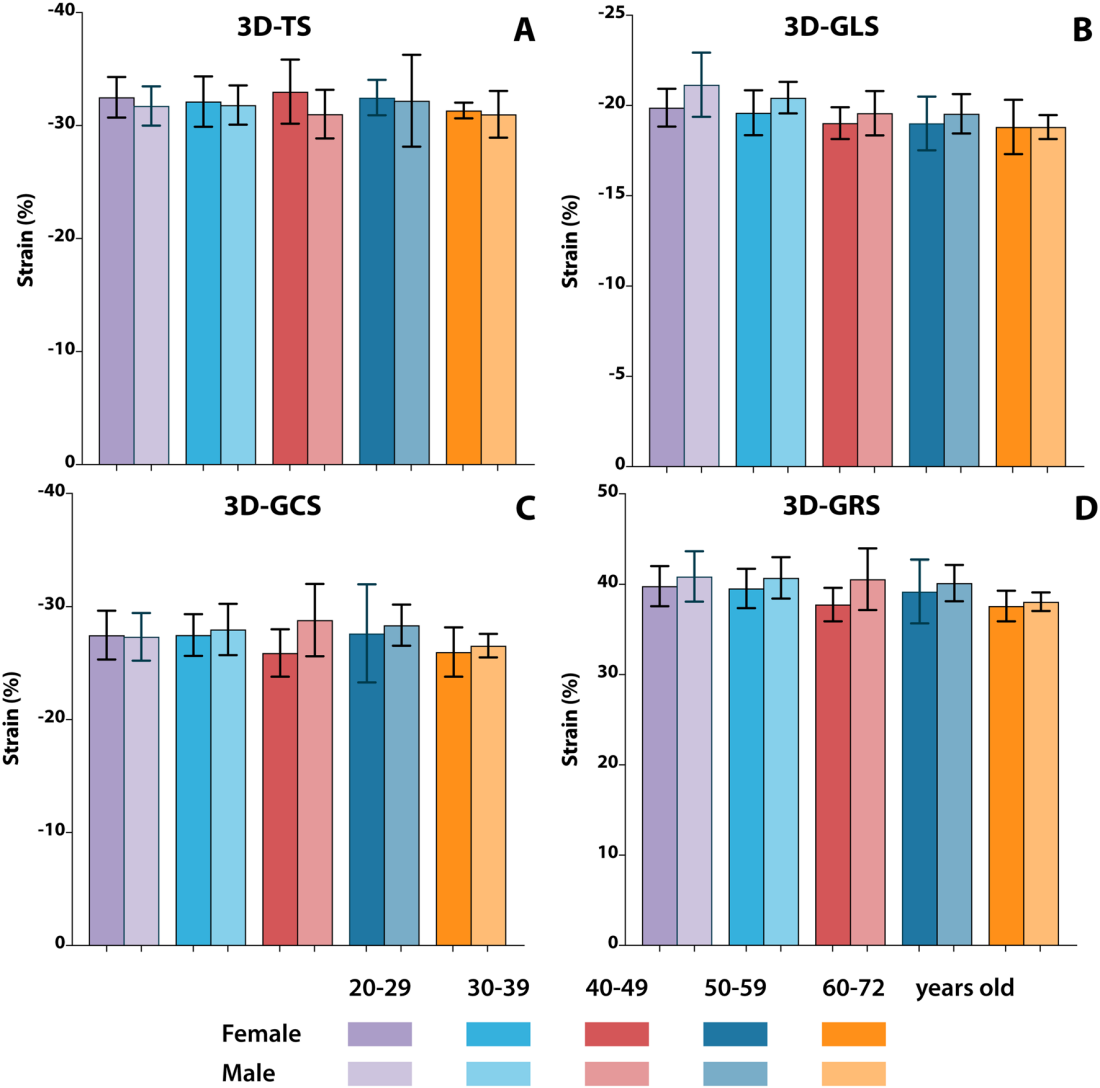
Distribution of 3D strain values in age and gender strata. *TS* tangential strain, *GLS* global longitudinal strain, *GCS* global circumferential strain, *GRS* global radial strain. Error bars represent 95% confidence interval of the mean

**Fig. 4:**
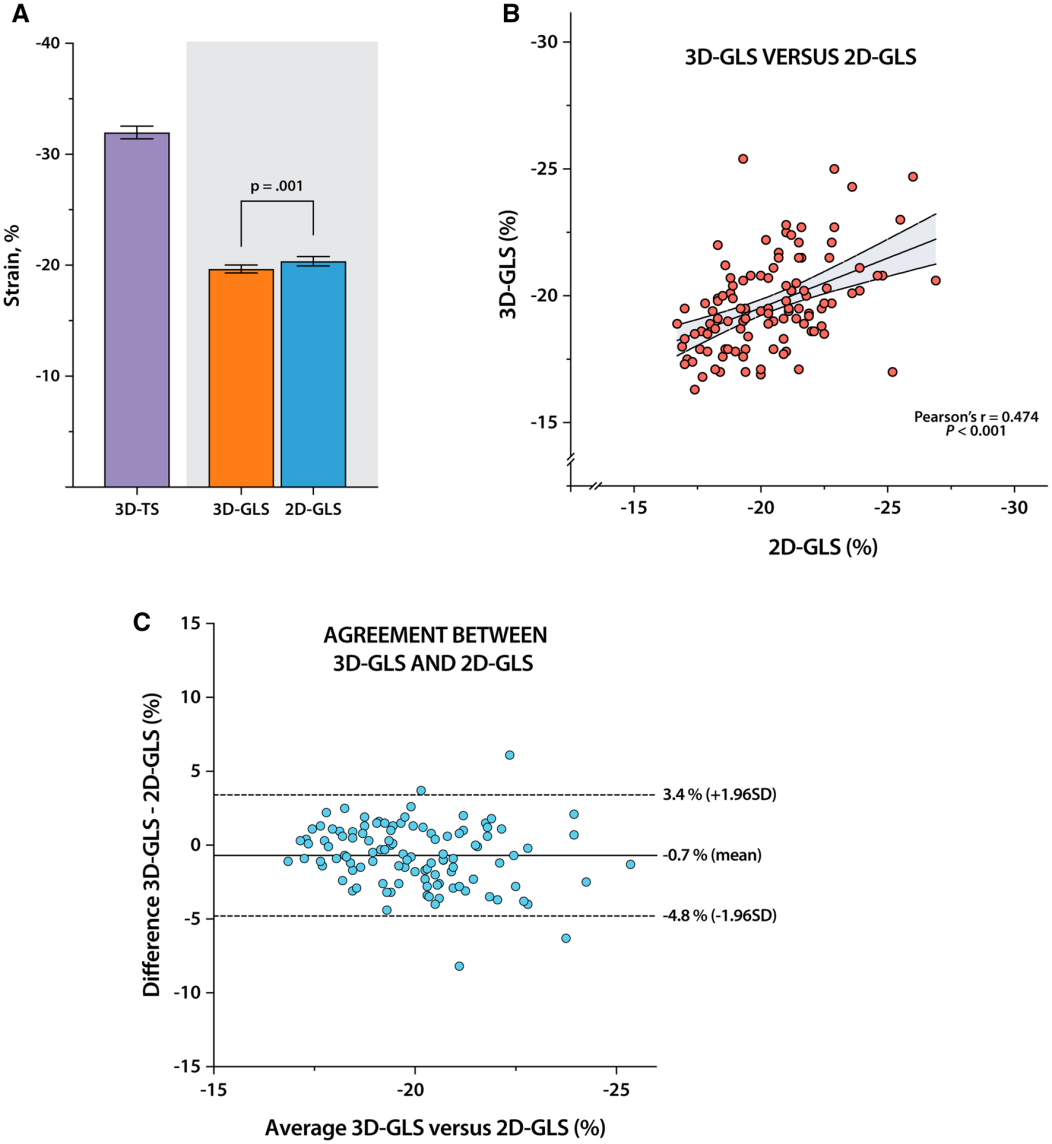
**a** Distribution of 3D-TS, 3D-GLS, 2D-GLS in the study population **b** Scatter plot graph demonstrating correlation of 3D-GLS and 2D-GLS measurements **c** Bland–Altman graph demonstrating agreement between of 3D-GLS versus 2D-GLS measurements. Error bars represent 95% confidence interval of the mean, *r* Pearson’s r coefficient, *TS* tangential strain, *GLS* global longitudinal strain

3D strain values are shown in Table 3, the distribution of the values in age and gender strata is shown in Fig. 3. Mean 3D-TS was − 32 ± 2.9% in the total study population. There were no significant gender-related differences with 3D-STE strain analysis. Among 3D strain parameters, only 3D-GLS decreased with increasing age strata (p = 0.024). 3D-LVEDV and 3D-LVESV were lower in female participants while 3D-LVEF did not differ between genders. The 3D-GLS values were lower compared with 2D-GLS values (− 19.7 ± 1.8% vs − 20.4 ± 2.2%, p = 0.001) (Fig. 4a). There was a statistically significant, but moderate correlation with a bias of − 0.7 ± 2.1 between 3D-GLS and 2D-GLS (r = 0.462, p < 0.*0*01) (Fig. 4b, c).

**Table 3:** Strain parameters by 2D-STE and 3D-STE in total population and distribution of the parameters according to sex and age strata

	Total	Sex			Age strata					
	n = 105	Female (n = 56)	Male (n = 49)	P	20–29 (n = 25)	30–39 (n = 22)	40–49 (n = 18)	50–59 (n = 22)	60–72 (n = 18)	P
3D strain parameters										
3D-TS (%)	− 32 ± 2.9	− 32.3 ± 2.8	− 31.6 ± 3	0.209	− 32.1 ± 2.8	− 32 ± 2.9	− 32 ± 3.3	− 32.4 ± 3.5	− 31.2 ± 1.7	0.783
3D-GLS (%)	− 19.7 ± 1.8	− 19.9 ± 1,7	− 19.3 ± 1.7	0.103	− **20.5 ± 2.3**	− **20 ± 1.6**	− **19.3 ± 1.4**	− **19.3 ± 1.8**	− **18.8 ± 1.3**	**0.024**
3D-GCS (%)	− 27.4 ± 3.3	− 27.8 ± 3.2	− 27 ± 3.4	0.194	− 27.4 ± 3.4	− 27.7 ± 3	− 27.3 ± 3.7	− 28.1 ± 3.9	− 26.3 ± 2	0.525
3D-GRS (%)	39.5 ± 3.5	40.1 ± 3.6	38.9 ± 3.2	0.08	40.3 ± 4	40.1 ± 3.3	39.2 ± 3.7	39.8 ± 3.7	37.9 ± 1.7	0.172
3D Twist (º)	12.5 ± 6.4	12.3 ± 6.1	12.8 ± 6.8	0.734	13 ± 6.9	12.1 ± 6	12.2 ± 6.2	14.3 ± 7	10.7 ± 5.9	0.508
3D Torsion (º/mm)	1.4 ± 0.7	1.5 ± 0.8	1.4 ± 0.7	0.518	1.4 ± 0.8	1.3 ± 0.7	1.3 ± 0.7	1.6 ± 0.9	1.3 ± 0.7	0.561
2D strain parameters										
2D-GLS (%)	− **20.4 ± 2.2**	− **21.1 ± 2.3**	− **19.5 ± 1.8**	** < 0.001**	− 20.7 ± 2.5	− 20.7 ± 2	− 20.2 ± 1.7	− 20.6 ± 2.6	− 19.2 ± 1.7	0.156
2D-MS CS (%)	− 28.9 ± 5.3	− 29.4 ± 4.9	− 28.2 ± 4.9	0.250	− **27 ± 4.5**	− **28.2 ± 4**	− **30.5 ± 6.4**	− **28 ± 4.6**	− **31.7 ± 6.5**	**0.028**
2D-MS RS (%)	48.5 ± 19.5	49.3 ± 19.3	47.6 ± 19.9	0.671	**40.5 ± 12.8**	**48.4 ± 19.4**	**62 ± 18.5**	**40.7 ± 17.8**	**55.9 ± 22**	** < 0.001**
3D volume parameters										
3D LVEDV (ml)	**142 ± 32**	**128 ± 22**	**159 ± 33**	** < 0.001**	148 ± 28	142 ± 26	149 ± 35	138 ± 23	133 ± 46	0.522
3D LVESV (ml)	**61 ± 16**	**54 ± 11**	**70 ± 17**	** < 0.001**	62 ± 15	60 ± 13	66 ± 17	60 ± 13	60 ± 22	0.763
3D LVEF (%)	57 ± 4	58 ± 4	56 ± 4	0.101	58 ± 4	58 ± 3	56 ± 5	57 ± 4	56 ± 2	0.124
2D volume parameters										
2D LVEDV (ml)	**120 ± 28**	**107 ± 21**	**136 ± 26**	** < 0.001**	123 ± 25	120 ± 29	127 ± 33	117 ± 35	115 ± 30	0.720
2D LVESV (ml)	**52 ± 15**	**44 ± 11**	**61 ± 15**	** < 0.001**	53 ± 15	51 ± 16	54 ± 16	50 ± 12	52 ± 18	0.954
2D LVEF (%)	**57 ± 5**	**58 ± 4**	**55 ± 4**	** < 0.001**	57 ± 5	58 ± 5	58 ± 4	57 ± 4	55 ± 5	0.419

Bold correspond to the parameters with values with a significant difference between comparison groups (P < 0.05)

Values are presented as mean ± SD

*3D* 3-dimensional, *2D* 2-dimensional, *GLS* global longitudinal strain, *GCS* global circumferential strain, *MS* mid-segment, *GRS* global radial strain

^*^The Pearson’s r coefficient and P values for correlation with respective 2D-STE parameters

^†^Correlation of 3D-GCS versus 2D-MS-CS

### Variables associated with 3D-TS and 3D-GLS

Results of the univariate and multivariate analyses are summarized in Table 4. In univariate analysis, lower end-diastolic intraventricular septum diameter (IVSd) (r = 0.229, p = 0.021), LVESV (r = − 0.419, p < 0.001) and LVEF (r = − 0.819, r < 0.001) were associated with 3D-TS. In a model consisting of age, systolic blood pressure, PWd, E wave, E’ wave amplitude and LVEF, only LVEF was independently associated with 3D-GLS (USβ = − 0.156, Sβ = − 0.346, R^2^ = 0.307, p = 0.001). Diastolic blood pressure demonstrated a strong correlation with systolic blood pressure, and E/A had a strong correlation with E wave amplitude. Thus, these 2 parameters were excluded from the model. Previous studies suggest that the effect of heart rate on myocardial strain is mainly via the changes in volumes and contractility. This parameter was also excluded from the model as a result. Multivariate analysis was not performed for 3D-TS, because the 3 predictors, IVSd, LVESV and LVEF correlated significantly with each other.

**Table 4:** Univariable and multivariable analyses of left ventricular 3D global strain and 3D global longitudinal strain

	3D-TS		3D-GLS				
	Univariate analysis		Univariate analysis		Multivariate analysis		
	r	p	R	*p*	USβ	Sβ	P
Age (years)	0.089	0.366	**0.285**	**.003**	− 0.003	− 0.027	.837
Sex (female)		0.209		.103			
Physical examination							
Body surface area (m^2^)	0.094	0.341	0.134	.174			
Systolic blood pressure (mmHg)	0.160	0.105	**0.291**	**.003**	0.019	0.153	.117
Diastolic blood pressure (mmHg)	0.199	0.042	**0.271**	**.005**			
*ECG*							
Heart rate (bpm)	0.105	0.287	**0.207**	**.034**			
QRS duration (ms)	− 0.035	0.719	0.018	.857			
Echocardiography, left ventricle							
IVSd	**0.229**	**0.021**	**0.216**	**.029**			
PWd	0.224	0.024	**0.229**	**.021**	0.003	0.002	.986
End-systolic dimension (mm)	0.105	0.288	0.063	.522			
End-diastolic dimension (mm)	− 0.019	0.844	− 0.123	.213			
E wave (m/s)	− 0.218	0.025	− **0.387**	** < 0.001**	− 0.124	− 0.011	.922
A wave (m/s)	− 0.104	0.291	0.015	0.883			
Deceleration time (ms)	− 0.098	0.322	− 0.002	0.983			
E' (cm/s)	− 0.160	0.106	− **0.412**	** < 0.001**	− 0.188	− 0.269	.054
E/A-ratio	− 0.035	0.724	− **0.259**	**0.008**			
E/E'-ratio	− 0.012	0.903	0.183	0.065			
LVEDV (mL)^a^	0.163	0.096	− 0.020	0.842			
LVESV (mL)^a^	− **0.419**	** < 0.001**	0.157	0.111			
LVEF (%)^a^	− **0.819**	** < 0.001**	− **0.477**	** < 0.001**	− **0.156**	− **0.346**	**.001**

Bold correspond to the parameters with values with a significant difference between comparison groups (P < 0.05)

*3D-TS* 3 dimensional tangential strain, *GLS* global longitudinal strain, *USβ* unstandardized β coefficient, *Sβ* standardized β coefficient, *LVIVSd* left ventricle interventricular septum thickness, *LVPWd* LV posterior wall thickness, *LVEDV* LV end-diastolic volume, *LVESV* LV end-systolic volume, *LVEF* LV ejection fraction

^a^volumes and LVEF in this value are measured by 3D-STE

### Intra-observer and inter-observer agreement

**Fig. 5:**
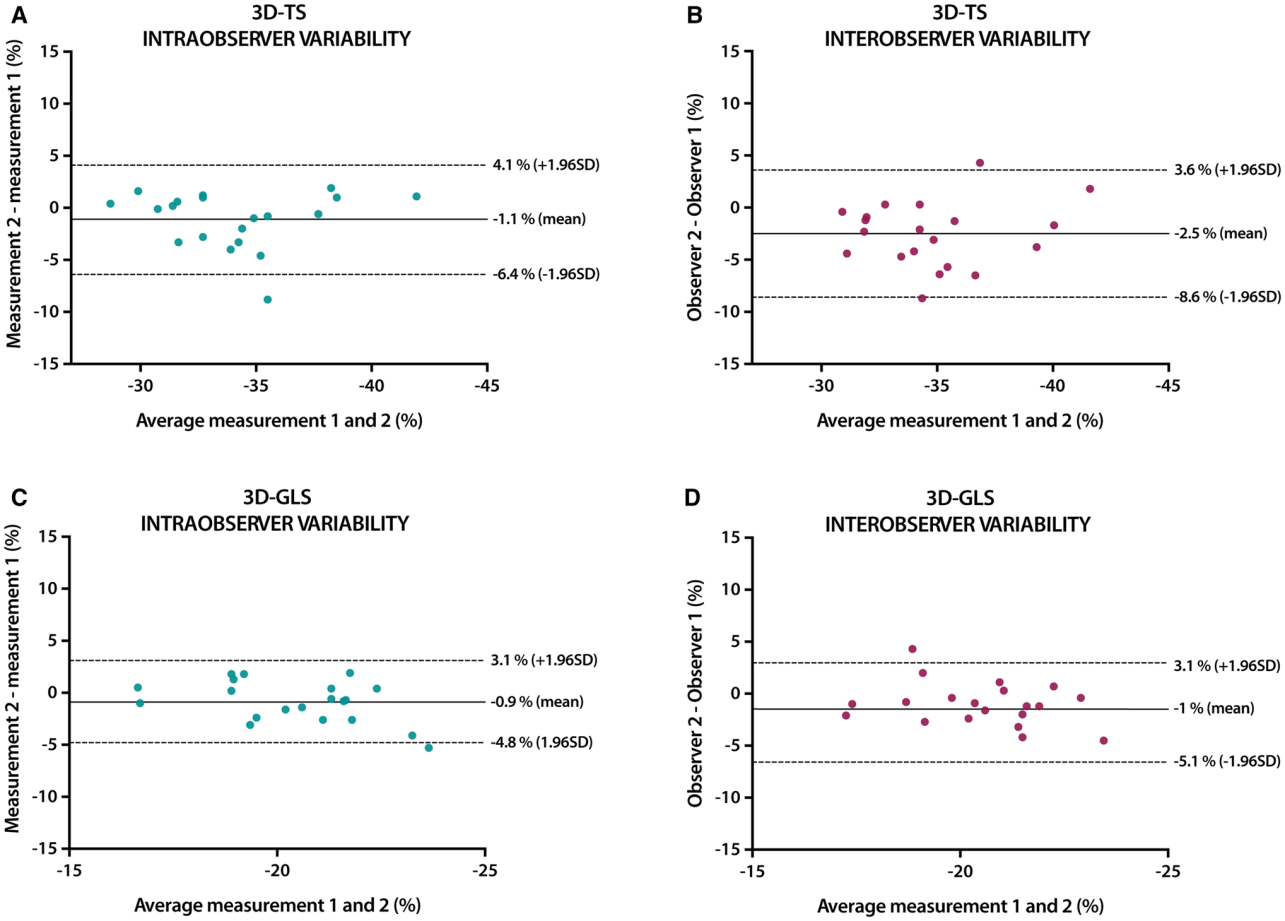
Intraobserver and interobserver analysis for 3D-TS (**a**, **b**) and 3D-GLS (**c**, **d**), respectively. *TS* tangential strain, *GLS* global longitudinal strain, *SD* standard deviation

The intra-observer variability was − 1.1 ± 2.7% and the inter-observer variability was − 2.5 ± 3.1% for 3D-TS. The intra and inter-observer variabilities for 3D-GLS were − 0.9 ± 2% and − 1 ± 2.1% (Fig. 5).

## Discussion

The current study presented normative values for 3D-STE strain variables for a healthy caucasian population. The feasibility of 3D-STE was lower than 2D-STE. The 3D-GLS values correlated significantly with 2D-GLS. LVEF was the only baseline characteristic that was associated with both 3D-TS and 3D-GLS.

### 3D-STE: normative data

The mean 3D-STE strain values were similar to published data on the same population [8], however lower than the multinational registries [5], despite similar BMI values. Normative ranges of 2D transthoracic echocardiography parameters were higher compared with the other populations in previous studies. This findings were explained with anthropometric characteristics, since Dutch people are the tallest of the world [9]. Larger body surface area with larger acoustic window might be expected to increase accuracy of the measurements. Conversely, larger lung tissue, larger BMI with resultant increase in subcutaneous adipose tissue might decrease the image quality and the accuracy of the strain measurements. Previously increased body size was found associated with decreased strain measurements using B-mode strain [14]. Population-specific genetic, structural or physiological characteristics other than anthropometric measures might also result in these differences.

The variability might alternatively result from the US-system or the STE analysis software. Gayat et al. demonstrated that US vendor (Toshiba versus Philips) was associated with variability in strain values [15]. Factors related with the US system such as probe specifications and image processing, and differences in temporal and spatial resolution of the system, as well as differences resulting from strain definitions (tangential vs area), region of interest selection (endocardial vs full thickness) and temporal definitions (ie. timing of the end-systole and end-diastole) are the potential sources of variability (Appendix 2) [16]. The EACVI-ASE Strain Standardization Task Force provides ongoing work in characterizing the inter-vendor variability and standardization of strain imaging with STE [17].

### 3D-STE versus 2D-STE

Along with the solution of geometric simplification and apical shortening, 3D-STE is equipped with the capability of producing novel 3D strain measures such as 3D-TS [4]. Nevertheless, limited temporal resolution is a well-known limitation of 3D-STE, when compared with 2D-STE. Recommended frame rate is 40 to 80 Hz for 3D-STE, although reported frame rates are usually lower [6]. Frame rates achieved with 3D-STE in our study were similar to the values obtained in previous studies but below the recommended range as well as the frame rates achieved by 2D-STE.

We compared measurement of GLS, the LV strain parameter with the most robust evidence, with 2D-STE and 3D-STE. 3D-GLS demonstrated significant correlation with 2D-GLS. The normative range of 3D-GLS was significantly lower than with 2D-GLS, which is consistent with the previous findings [18]. In a recent study apical foreshortening resulted in up to 23% overestimation in 2D-STE [3]. Lower 3D-GLS values compared with 2D-GLS in this study might be the result of a more accurate localization of the apex. We suggest that 3D-GLS could be implemented in measurement of GLS, but the validated reference ranges for 2D-GLS should not be used interchangeably with 3D-GLS.

### Relationships of 3D-STE with age, sex and LVEF

Previous studies have found an age-related decrease in 3D-GLS [8, 19]; however, large scale studies and meta-analyses failed to confirm these patterns [20]. In our study, 3D-GLS decreased, while 3D-GCS and 3D-GRS did not change with increasing age. 3D-GLS was not associated, and the remaining 3D strain parameters were weakly associated with age in the recent NORRE study [5]. Different studies have shown a decreased [21], or increased [19] 3D-GCS and 3D-GRS values with increasing age.

LVS values has been found higher in women in previous studies [5]. This is usually attributed to higher LVEF in women [9]. However, a recent meta-analysis failed to demonstrate this difference [6]. The differences in 3D strain values were not statistically different between genders in this study. This finding paralleled similar 3D-LVEF values between genders. Lack of replicability in the age and gender-related changes among previous studies and also the current one has been attributed to the complex interplay between population-specific and vendor-specific factors. It is suggested that gender-related differences in LV strain was attributed to differences in body size between genders using B-mode strain measurements. Populations with closer body size ranges between genders might have closer LV strain values [14]. Breast tissue in women sometimes makes localization of the true apex difficult. Resulted foreshortening might also result in higher values in women [3].

Another possible factor underlying this variability is sample size. Especially for cohorts that consist of healthy individuals, distribution range of both strain and volumetric parameters is narrower. As a result, retrieving significant test results would require considerably large sample sizes in such studies. The statistically nonsignificant but higher 3D-TS and 3D-GLS and 3D-LVEF values in women, and lower 3D-TS, 3D-LVEDV and 3D-LVEF with increased age might be associated with these last 2 factors. The large international registries [5] as well as studies on populations with different ethnic background, will delineate ethnic as well as age and gender-related differences with regard to myocardial deformation patterns.

LVEF was the only factor associated with both 3D-TS and 3D-GLS. This finding is in accordance with the findings from other studies in healthy individuals [5]. When 3D-STE and 2D-STE are compared, 3D-TS and 3D-GLS correlated stronger with LVEF compared with 2D-GLS in previous studies [22]. GLS is a sensitive marker of LV global systolic function and might be impaired in subclinical cardiac damage, when LVEF is within normal limits [8]. However, in healthy individuals without cardiovascular disease, GLS is expected to correlate better with LVEF. We demonstrated that 3D-TS and 3D-GLS are sensitive to changes in LVEF values within normal range in healthy adults.

### Feasibility and reproducibility

The patients for the current study were initially enrolled for 2D-STE. The feasibility of 3D-STE reported herein might reflect the real-world experience better as a result. Yet, the feasibility of 3D-STE in our study was higher than some of the reported values as low as 71% [23]. Higher BMI is associated with decreased transthoracic echocardiography image quality. Resultant decreased feasibilty was demonstrated in the current study. Lower E wave amplitude in the 3D-STE nonfeasible group might be associated with increased BMI [24].

The reproducibility of 3D-TS and 3D-GLS was acceptable [8]. The manual corrections performed during the endocardial tracing is an important source of variability for 3D-STE. Implementation of fully automated border tracking and machine-learning algorithms by the 3D-STE systems will potentially eliminate the problems related with reproducibility in the future [25].

### Limitations of the study

The software platform used for 3D-STE only allows endocardial strain analysis. 3D-STE analysis package didn’t allow exclusion of non-tracking or poorly-tracking segments. An approach focusing on global average values was preferred as a result. Demonstration of the intervendor variability would have had incremental role in this study. However, we think that only inter-vendor agreement, but not the validity of a certain vendor, could be assessed in such a study, since there is no currently available noninvasive gold standard that the in-vivo accuracy of the available platforms could be tested against.

## Conclusion

Age and sex-related changes as well as normative values were provided in this single US system, single analysis software study on healthy adult Caucasians. 3D-STE demonstrated reasonable feasibility and excellent agreement with LVEF. 3D-STE should be implemented in clinical evaluation of the LV function. The findings herein support interpretation of 3D-STE based myocardial deformation with vendor- and population-specific reference ranges. The 3D strain values should not be used and interpreted interchangeably with 2D strain values.

## Data Availability

The data is available and permission might be sought per reasonable request.

## Appendix 1

See Table 5

**Table 5:** Characteristics of the study population, according to age and gender strata

Characteristic	Sex		p	Age group (years)					p
	Female	Male		20–29 n = 32	30–39 n = 28	40–49 n = 28	50–59 n = 31	60–72 n = 28	
Sex, female	–	–		16 (50%)	14 (50%)	14 (50%)	16 (50%)	14 (50%)	
Age (years)	45 ± 14	44 ± 14	.581	26 ± 2	**35 ± 3**	**44 ± 3**	**54 ± 3**	**64 ± 3**	** < .001**
Current smoker	8 (12%)	5 (8%)	.569	2	1	4	5	1	.329
Physical examination									
Body mass index (kg/m^2^)	**23.5 ± 3**	**25.2 ± 3.3**	**.002**	**22.3 ± 2.1**	**24 ± 3.4**	**24.6 ± 3.7**	**25.3 ± 2.8**	**25.8 ± 3.2**	** < .001**
Body surface area (m^2^)	**1.76 ± 0.12**	**2.03 ± 0.15**	** < .001**	1.83 ± 0.17	1.89 ± 0.18	1.92 ± 0.22	1.91 ± 0.18	1.90 ± 0.19	.370
Systolic blood pressure (mmHg)	**123 ± 12**	**131 ± 16**	**.001**	**124 ± 13**	**131 ± 10**	**123 ± 12**	**130 ± 15**	**136 ± 17**	** < .001**
Diastolic blood pressure (mmHg)	**77 ± 9**	**82 ± 9**	** < .001**	**76 ± 8***	**78 ± 7**	**79 ± 9**	**83 ± 11**	**83 ± 8**	**.005**
ECG									
Sinus rhythm									
Heart rate (bpm)	63 ± 8	60 ± 10	.066	61 ± 11	60 ± 8	62 ± 9	65 ± 10	65 ± 10	.372
PR interval (ms)	**155 ± 22**	**163 ± 21**	**.029**	**155 ± 23**	**150 ± 21**	**156 ± 24**	**159 ± 16**	**172 ± 18**	** < .001**
QRS duration (ms)	**92 ± 7**	**100 ± 9**	** < .001**	96 ± 8	97 ± 9	97 ± 9	94 ± 10	97 ± 9	.849
Echocardiography, left ventricle									
End-diastolic dimension (mm)	**44 ± 4**	**46 ± 4**	**.002**	46 ± 3	47 ± 3	45 ± 4	45 ± 4	44 ± 5	.051
End-systolic dimension (mm)	**27 ± 4**	**29 ± 4**	**.026**	28 ± 3	28 ± 3	28 ± 5	28 ± 4	28 ± 6	.990
E wave (m/s)	**0.72 ± 0.17**	**0.66 ± 0.14**	**.006**	**0.80 ± 0.16***	**0.75 ± 0.16**	**0.66 ± 0.15**	**0.65 ± 0.10**	**0.59 ± 0.13**	** < .001**
A wave (m/s)	0.51 ± 0.16	0.48 ± 0.14	.229	**0.39 ± 0.14***	**0.43 ± 0.08**	**0.47 ± 0.10**	**0.56 ± 0.12**	**0.62 ± 0.17**	** < .001**
Deceleration time (ms)	186 ± 31	194 ± 49	.191	**177 ± 29**	**181 ± 32**	**185 ± 29**	**194 ± 31**	**216 ± 63**	**.001**
E' (cm/s)	9.5 ± 2.7	9.4 ± 2.4	.680	**12.4 ± 1.8**	**10.4 ± 1.6**	**9.2 ± 1.6**	**8.2 ± 1.8**	**6.8 ± 1.7**	** < .001**
E/A-ratio	1.6 ± 0.7	1.5 ± 0.6	.443	**2.3 ± 0.8**	**1.8 ± 0.4**	**1.4 ± 0.4**	**1.2 ± 0.3**	**1.0 ± 0.3**	** < .001**
E/E'-ratio	**7.9 ± 2**	**7.3 ± 1.7**	**.039**	**6.4 ± 1.3**	**7.3 ± 1.5**	**7.3 ± 1.7**	**8.1 ± 1.4**	**9.0 ± 2.4**	** < .001**
End-diastolic volume (mL)	**103 ± 18**	**134 ± 22**	** < .001**	117 ± 23	119 ± 23	125 ± 30	116 ± 21	116 ± 30	.704
End-systolic volume (mL)	**39.9 ± 10**	**55 ± 12**	** < .001**	47 ± 11	47 ± 13	51 ± 12	45 ± 12	48 ± 16	.462
End-diastolic volume / BSA (mL/m^2^)	**58 ± 9**	**66 ± 9**	** < .001**	64 ± 9	63 ± 10	64 ± 11	60 ± 8	61 ± 12	.492
End-systolic volume/BSA (mL/m^2^)	**23 ± 5**	**27 ± 5**	** < .001**	25 ± 5	25 ± 6	26 ± 5	23 ± 5	25 ± 7	.370
Ejection fraction, biplane (%)	**62 ± 5**	**59 ± 4**	** < .001**	60 ± 4	61 ± 5	59 ± 4	62 ± 5	59 ± 4	.099

Bold correspond to the parameters with values with a significant difference between comparison groups (P < 0.05)

Values are presented as n (%) or mean ± SD

*E* peak mitral inflow velocity at early diastole, *A* peak mitral inflow velocity at late diastole, *E* early diastolic annular myocardial velocity, *BSA* body surface area

.

## Appendix 2

See Table 6

**Table 6:** Probable sources of intervendor variability in speckle tracking echocardiography (Adapted from 16)

Modality and US system related (2D-STE versus 3D-STE or inter-vendor)	• Technical differences in image acquisition and processing • Spatial and temporal resolution
Software-related	• Strain definitions (eg. tangential vs area strain) • Calculation of global values (peak vs simultaneous) • Layer preference (only endocardial versus fixed thickness ROI vs adjustable thickness ROI tracking) • Definition of end-diastole and end-systole (ECG, valvular closure/openings, inflow/outflow)

*3D-STE* three-dimensional speckle tracking echocardiography, *ROI* region of interest

.
